# Editorial

**Published:** 1864-06

**Authors:** 


### Editorial.

---

Illinois State Medical Society.—The recent Annual Meeting of the Illinois State Medical Society in this city was well attended, and afforded a season of more than usual interest. A larger number of interesting papers and reports were read than at any former meeting for several years.

All the proceedings and discussions were harmonious and cordial; the visits to the Medical Colleges and Hospitals interesting and profitable; and the social union of the members of the State Society with those of the Chicago City Society, at the residence of the Permanent Secretary, on Wednesday evening, was an occasion of high social enjoyment. We give, in another part of the EXAMINER, a full record of the proceedings, and also the first half of the report of the Committee on Practical Medicine. We publish this part of the report thus early on account of the general interest felt in the subject of Cerebro-Spinal Meningitis or Spotted Fever.

Other papers in relation to the same subject will be published in the next number of the EXAMINER; while the remaining part of the report, relating to the improvements in Practical Medicine, will be deferred to the August number.

---

THE following interesting and important communication was received too late for insertion in the proper place under the head of original articles:—

### CLEFT PALATE, CONGENITAL AND OTHERWISE. THEIR REMEDIES.

Having recently spent some time in examining cases of palatine fissure, and the various means adopted for their treatment in Europe, I would add my testimony in view of the opportunities which I have enjoyed, to that which may soon appear upon the same subject, in favor of an appliance invented by Dr. N. W. KINGSLEY, of New York City.

It has been successfully tested in a number of cases, for about four years, and may, therefore, be safely regarded as considerably in advance of those fixtures heretofore adopted to remedy this most disagreeable defect. While in Europe, I sought out and examined many of the patients who had been operated upon for fissure, particularly those for whom artificial "obturators" had been made; conversed with them while their obturators were in place; and then caused their removal in order that I might judge of the amount of benefit that had been derived from them. I found, however, in no one of these cases an apparatus that exactly accomplished the desired object.

Without going into the history of these cases, or their treatment; so far as I have been able to ascertain, no operation for fissure, whether surgical or consisting of a mechanical appliance, has until now been more than partially successful; and many cases, now amenable to treatment, have been regarded as entirely remediless.

Before giving a description of the appliance, I will briefly rehearse the indications to be fulfilled in the adaptation of an artificial velum.

There is needed such a substitute for the soft palate and uvula, as shall be under the control of the muscles acting upon those parts; and this is quite possible, inasmuch as the essential parts of the palato glossus muscles and the palato pharyngei are almost always found remaining, both in cases of congenital fissure, and in those of accidental lesions of the palatine organs.

In the first place, this will allow of the elevation and depression of the artificial substitute, at will, so that the voice can be emitted through the oral or nasal orifice, or both, as may be required; and this is the main desideratum, for it is comparatively easy to provide for the necessities of mastication and deglutition by a simple covering to the orifice sufficiently resisting to withstand the pressure. Secondly, it should furnish such an adaptation as shall be at the same time accurate, impervious to the passage of liquids, or the breath in speech, and yet shall exert no pressure upon the surrounding parts to cause absorption or atrophy of the palatine or nasal organs. Thirdly, there is needed a substance that shall be susceptible of the motions of the soft palate, and shall not so weary the remaining muscles in the performance cf their office (while this fixture is "in situ,") as to be impracticable.

These indispensible indications are fulfilled in the artificial velum under consideration, in the last particular by being made of vulcanized soft rubber, so constructed as to be movable upon and over itself, independent of its softness, elasticity, and generally non-irritating character, being thus susceptible of the contraction, expansion, elevation, and depression of the velum, in an extraordinary degree.

The third indication is admirably fulfilled by having a flange perfectly adapted, over the edges of the orifice, both superiorly and inferiorly, through the medium of a plaster of Paris impression of the parts, which is taken with the plaster about the consistency of cream, thus ensuring absolute accuracy.

The nature of the rubber itself, together with its lightness and its necessarily yielding form, (due to the manner of its construction) ensure against any undue pressure.

Finally, its perfect adaptation both to the anterior superior portion of the fissure, as well as to the posterior inferior and lateral parts, by means of the double flange of which mention has before been made, secures the control of the two pairs of muscles above mentioned, and generally of the levator palati also.

With these advantages in his favor, together with the additional one of the fixtures being held firmly in its place by means of gold attachments to the teeth, the patient proceeds to *learn* to talk, by learning the proper uses of the long disused or misused pharyngeal muscles, and the success of patients treated in this manner over those treated in any other is truly remarkable. One point more I will mention: by adopting this means of remedying congenital palatine defects, the operation can be performed much earlier than by the older methods, inasmuch as in the growth of the patient the fissure is enlarged longitudinally, and that enlargement can be easily provided against in the construction of the appliance; and thus by providing for the defect at an early period in life, much better results are obtained than by waiting until misuse has resulted in a permanent tendency to an imperfect pronounciation.

E. A. BOGUE, D.D.S.

*Chicago, May,* 1864.

---

Obstinate Constipation.—M. Homolle has found the following powder efficacious in two cases, where obstinate constipation had raised the question of operation for artificial anus:—Powdered strychnine, one-fiftieth of a grain; powdered nux vomica, one-fifth of a grain; calcined magnesia, six grains; mix. One powder a day at first, then two, and finally three per diem. In both cases the bowels were moved, and the symptoms of suspected internal strangulation disappeared.

